# Supplementary material for: Influence of relay intercropping of barley with chickpea on biochemical characteristics and yield under water stress
Source: PLoS One. 2023 Jun 8;18(6):e0273272. doi: 10.1371/journal.pone.0273272 (PMC10249810; doi:10.1371/journal.pone.0273272)
Supplement: Attachment — Submitted filename: Response to Reviewers.docx [file pone.0273272.s002.docx]

**Dear Editor of Plos One**

I sent the **tracked-change revision** of our manuscript entitled ''Influence of relay intercropping of barley with chickpea on biochemical characteristics and yield under water stress'' (PONE-D-22-21858) as well as **Response to Reviewers** by attachments. Also, I replied to reviewer's comments as below:

Additional Editor Comments:
Dear authors,I agree with both reviewers that the ms is interesting but badly handled by the statistical point of view. In particular, the statistical analysis is not replicable and application of a LSD post hoc to such a complex design will bring you to high chances of false positive results.

**Author response:** I used Tukey test instead of LSD throughout the manuscript.

In addition to the suggestion above, I suggest you to change the sentence "Means sharing the same letter do not differ significantly based on LSD (p≤0.05) test." to "Means with a letter in common should not be considered different according to the post-hoc test at p≤0.05".

**Author response:** See all of the tables and figures., please.

Lastly, I mind you that Plos requires for the row data to be published. You should upload at least the means and standard deviations or errors (to be specified along the number of replicates) in the supplementary materials or other repository and indicate it in the ms.

**Author response:** Means data ±SE are given throughout the manuscript.

**Comments to the Author**

1. Is the manuscript technically sound, and do the data support the conclusions?

The manuscript must describe a technically sound piece of scientific research with data that supports the conclusions. Experiments must have been conducted rigorously, with appropriate controls, replication, and sample sizes. The conclusions must be drawn appropriately based on the data presented.

Reviewer #1: Partly

Reviewer #2: No

**Author response:** See the text, please. The conclusion section has been revised, as well.

2. Has the statistical analysis been performed appropriately and rigorously?

Reviewer #1: I Don't Know

Reviewer #2: No

**Author response:** The statistical analysis changed and cropping system in each irrigation regime and year compared, separately. Also, the means were compared by Tukey's test.

3. Have the authors made all data underlying the findings in their manuscript fully available?

The [PLOS Data policy](http://www.plosone.org/static/policies.action#sharing) requires authors to make all data underlying the findings described in their manuscript fully available without restriction, with rare exception (please refer to the Data Availability Statement in the manuscript PDF file). The data should be provided as part of the manuscript or its supporting information, or deposited to a public repository. For example, in addition to summary statistics, the data points behind means, medians and variance measures should be available. If there are restrictions on publicly sharing data—e.g. participant privacy or use of data from a third party—those must be specified.

Reviewer #1: Yes

Reviewer #2: Yes

4. Is the manuscript presented in an intelligible fashion and written in standard English?

PLOS ONE does not copyedit accepted manuscripts, so the language in submitted articles must be clear, correct, and unambiguous. Any typographical or grammatical errors should be corrected at revision, so please note any specific errors here.

Reviewer #1: No

Reviewer #2: No

**Author response:** The manuscript has been revised. See the text, please.

**Reply to Reviewer #1:** While the research carried out might bring some valuable insights into the intercropping (and relay cropping) performance under water stress conditions, the manuscript presents several weaknesses that must be addressed. I provide some general comments for each part of the manuscript in this section:

- General: the English level of the manuscript is not adequate for a scientific manuscript. There are several grammar mistakes, misspellings, word choices and sentence constructions that make the text unintelligible at some points.
  **Author response:** The English level of the manuscript has been improved.
- Introduction: the ideas expressed are not well connected. I lacked some references and explanations relating to the relationship between pigment contents and crop performance and how intercropping affects these variables. **Author response:** Some references were added to this section. See the Introduction section, please.
- M&M: please, consider the size of plots in future research, 3x2 m is quite a small size for intercrop trials. Plots of this size tend to overestimate the yield that a farmer would get in a commercial field.

**Author response:** Yes. You are right.
You do not state the application field of your research: horticultural, field crops, etc. If field cropping is the target of your research, the use of sowing machines should be considered rather than hand sowing, as the latter does not represent the conditions a farmer might face in a field.

**Author response:** The relay intercropping system is more used for smallholder farmers and traditional agriculture in dry and tropical areas for most plants. Therefore, in this system, most of the plants are cultivated manually [2, 32].
The statistical analyses are not described in sufficient detail to be replicated by other researchers or to correctly interpret the results.

**Author response:** The statistical analysis changed and each irrigation regime and year compared, separately. Also, the means were compared by Tukey test.

Results: The result display is quite poor. The significative triple interaction in all the variables led to several Figures and Tables that are quite difficult to comprehend from an external reviewer. In addition, the result section does not provide clear and key results of this research.

**Author response:** Data was analyzed by SAS software 2012 (version 9.4) and the means were compared Tukey test at 0.05 probability level (p≤ 0.05). Because of significant effect of year × irrigation regime × cropping system on considered traits, the data of two years for barley and chickpea presented, separately. This section revised, again.

Discussion: the English level in which the discussion is written does not allow an objective evaluation of it, as some mistakes (grammar or word choice) can lead to incorrect judgement.
**Author response:** See the Discussion section, please.


Conclusions: some results are summarized in this section, but no clear conclusions are stated.

**Author response:** The Conclusions section has been revised.

**Reply to Reviewer** #2: The manuscript “Influence of relay intercropping of barley with chickpea on biochemical characteristics and yield under water stress” aimed to investigate the effects of water stress on barley-chickpea relay intercropping. The evaluation focused both on agronomic performance and biochemical parameters of crops.
On the whole, the topic of this paper is very interesting. Despite the effects of the co-cultivation between cereals and grain legumes on biochemical aspects of crops have been rarely investigated in previous study on intercropping, they are fundamental for better understand the interactions between crops in a more diversified cropping system. Therefore, I appreciate the novelty of this manuscript.

The introduction provides sufficient background, even if the number of relevant references can be increased. The research is design appropriately and methods are adequately described (except for the statistics section). I’m convinced that authors of this manuscript have extremely interesting data however they failed to valorize them in this manuscript. In fact, the results are not clearly presented, a lot of details are missing, and the manuscript is very difficult to read. Moreover, results can be misinterpreted due to the inappropriate statistical analysis.
**Author response:** In the introduction section, some new refences were added. Also, data was analyzed by SAS software 2012 (version 9.4) and the means were compared Tukey test at 0.05 probability level (p≤ 0.05). Because of significant effect of year × irrigation regime × cropping system on considered traits, the data of two years for barley and chickpea presented, separately.

- General comments:
  English language and style: The manuscript is not easy to read, an extensive editing of English language and style is required.

**Author response:** The English level of the manuscript has been improved.

- Statistical analysis: statistical analysis needs to be revised. I have several concerns related with the data presented in table 5, 6, 7, 8 and figures. Standard errors are missing everywhere, and unit of measures are often wrong. Please see specific comments below.
  Data presentation: Tables and figure needs to be deeply revised according to the revised statistical analysis.

**Author response:** The Standard errors were added in all of the tables and figures. Also, unit of measures has been checked, again.

Specific comments:
Line 47: Please add a reference “…another crop (cit).

**Author response:** See the text, please.
Line 53: Please add a reference “…of the world (cit).

**Author response:** See the text, please.
Line 53: Please add a reference “…final yield (cit).

**Author response:** I didn't find this sentence in line 53.
Line 84: “At the best of our knowledge” instead of “Until now”.

**Author response:** See the text, please.

Line 98: Please add reference defining the soil type e.g “Jahn R, Blume H, Asio V, Spaargaren O, Schad P (2006) Guidelines for soil description, 4th edn. FAO, Rome”.

**Author response:** See the text, please.

Line 126: Please, define the plowing depth.

**Author response:** The plowing depth was 30 cm.
Line 129: Define inter-rows space.

**Author response:** The inter-row space between barley and chickpea was 30 cm.
Line 144: for barley and chickpea the effective root zone is around 20 -25 cm, why did you consider 90 cm?

**Author response:** The effective root zone is the depth within which most crop roots are concentrated, which was estimated as ∼50–100 cm for barley and as ∼60–70 cm for chickpea (Fan et al., 2016). Thus, with respect to monitor the soil water content at root zone the soil water content was traced in each plot at 30 cm depth down to 90 cm, gravimetrically.

Table 3 and 4: In order to improve the tables readability, please define the meaning of I and C in the caption.

**Author response:** Irrigation regimes (I) included of normal irrigation and cutting off irrigation at full anthesis stage of barley and cropping system (c) consisted of sole and delay intercropping of barley and chickpea in December and January. See the text, please.
Lin 147: Is it a common practice irrigating barley in Iran? Or it is just an experimental practice?

**Author response:** Yes. it is a common practice in barley. Unfortunately, in some years, there is no considerable rainfall in March to May when the water requirement of safflower increased to complete the seed filling period (Table 2). These conditions usually are typical in sought of Iran which has dry and hot spring season. With respect to occurrence of rain fall in the cool season, the farmers have to irrigate the crop after anthesis.
Line 311: “mg. mg. g-1 “is not the correct annotation for the unit of measure. The correct annotation is “mg · g-1”. Please check all the unit of measure in the text and SE should be also included.

**Author response:** All of the units were checked throughout the manuscript.
Everywhere in the text: please, you should include standard error (SE) when you report data in the text.

**Author response:** See the text, please.

TABLES
Table 1:
• “Soil organic matter (SOM)” instead of “Organic content”

**Author response:** See the text, please.

• I would express N as g/kg.

**Author response:** That's converted to g/kg in the Table 1.
• P, is it total P or Olsen P? Same for the nitrogen, which kind of protocol did you follow for the Nitrogen determination? Kjeldahl method? Please add this information in table caption.

**Author response:** P content of the soil is Olsen P and N content of the soil was determined by the Kjeldhal method.

Table 5: You mentioned that “year” and “irrigation regime” are significant factors. In this case you should perform the statistical analysis separately for each year and for each irrigation regime. In the table you have assigned letters of significance in the wrong way. Please perform a post hoc separately for year and irrigation regime and eventually assign capital letter for indicating differences between years. You should include standard error (SE).
Table 6: see comments above
Table 7: see comments above
Table 8: see comments above

**Author response:** In all of the tables, each irrigation regime and year were analyzed, separately. Means with common capital letters in each irrigation regime within two years and means with common lowercase letters in each irrigation regime and year indicate no significant difference based on Tukey's test at p≤0.05. Vertical bars represent ±SE.

Line 362: “Unit. mg-1 protein” “Unit· mg-1 protein”.

**Author response:** That's converted to Unit/mg protein throughout the manuscript

FIGURE
Figure 1: You should perform post-hoc of your model separately for 2017 and 2018. Use lowercase letter for 2017 and uppercase letter for 2018. SE are missing, please provide them. In case that year is not a significant factor you could present data as average between 2017 and 2018 for each of the treatments. Another option can be present data on 2017 and 2018 in two separate graphs.

Same for figure 2, 3, 4, 5, 6, 7.

**Author response:** In all of the figures, data for each year was presented, separately.

**Sincerely,**

**Corresponding author**
