# Supplementary material for: Influence of relay intercropping of barley with chickpea on biochemical characteristics and yield under water stress
Source: PLoS One. 2023 Jun 8;18(6):e0273272. doi: 10.1371/journal.pone.0273272 (PMC10249810; doi:10.1371/journal.pone.0273272)
Supplement: Attachment — Submitted filename: Response to Reviewers.docx [file pone.0273272.s003.docx]

Dear Dr. Sergio Saia

Editor of Plos One

I sent the tracked-change revision of our manuscript entitled ''Influence of relay intercropping of barley with chickpea on biochemical characteristics and yield under water stress'' (PONE-D-22-21858) as well as Response to Reviewers by attachments. Also, I replied to reviewer's comments as below:

**Additional Editor Comments:**

Deal authors, one of the 2 former reviewers accepted to re-review your ms. I frankly have to say that I almost agree with the reviewer. In particular, the english language can be improved by yourself trying to use simpler sentences and making a general polishing of the work. So far, my greatest worry is about the statistical analysis and the reviewer pointed to the need of ensuring its reliability, by suggesting the GLMM (which, by the way, can also handle non normal distributed data o residues and few replications). I please exhort you to carefully take into account that the statistical analysis should be done reducing the false positive and false negative results, with a certain degree of protection of the results given by proper statistics (e.g. Benjamini and Hochberg or Tukey-Kramer, etc.)

**Author response:** The model used for variables in analysis of variance was fix model for all variables including year, irrigation regime and cropping system. In order to check the normality distribution of data, Kolmogorov-Smirnov and Shapori-Wilk tests were used and the skewness and kurtosis indices of data proved that the distribution of data was normal. F-test was carried out to check the equality of variances. The residuals of model were also normal using q-q plot. By the way, most of the researchers use 3 replicates and is often enough for such field experiments. Data was analyzed by SAS software 2012 (version 9.4) and the means were compared by Tukey-Kramer test at 0.05 probability level (p≤ 0.05).

Also, The English language of the manuscript has been improved, again. See the text, please.

Regarding the statistics, please pass over to the indication of the rev. regarding "Values with different letters are not significantly different at 0.05 confidence level." The correct statement is "Values with a letter in common should not be considered different at 0.05 probability.", which poses an additional degree of protection of the results.

**Author response:** It's done in the text.

Is the manuscript technically sound, and do the data support the conclusions?

The manuscript must describe a technically sound piece of scientific research with data that supports the conclusions. Experiments must have been conducted rigorously, with appropriate controls, replication, and sample sizes. The conclusions must be drawn appropriately based on the data presented.

Reviewer #2: Partly

3. Has the statistical analysis been performed appropriately and rigorously?

Reviewer #2: No

**Author response:** See the text, please.

4. Have the authors made all data underlying the findings in their manuscript fully available?

The [PLOS Data policy](http://www.plosone.org/static/policies.action#sharing) requires authors to make all data underlying the findings described in their manuscript fully available without restriction, with rare exception (please refer to the Data Availability Statement in the manuscript PDF file). The data should be provided as part of the manuscript or its supporting information, or deposited to a public repository. For example, in addition to summary statistics, the data points behind means, medians and variance measures should be available. If there are restrictions on publicly sharing data—e.g. participant privacy or use of data from a third party—those must be specified.

Reviewer #2: Yes

5. Is the manuscript presented in an intelligible fashion and written in standard English?

PLOS ONE does not copyedit accepted manuscripts, so the language in submitted articles must be clear, correct, and unambiguous. Any typographical or grammatical errors should be corrected at revision, so please note any specific errors here.

Reviewer #2: No

**Author response:** The English language of the manuscript has been improved, again. See the text, please.

6. Review Comments to the Author

Please use the space provided to explain your answers to the questions above. You may also include additional comments for the author, including concerns about dual publication, research ethics, or publication ethics. (Please upload your review as an attachment if it exceeds 20,000 characters)

Reviewer #2: The authors mentioned that the English has been revised in the manuscript but it is seems not: the paper is difficult to read, especially for the results section. Moreover, there are too many tables and figures. I would suggest removing some tables or move them in supplementary material and to add relevant information in the main text. Figures 3, 4 and 5 for instance can be merged in 1 graph. I still have some concerns about statistical analysis (see specific comments below). I’m convinced that your data and research topic is interesting however, I would suggest the authors for an extensive English check otherwise the MS could be not considered for the publication.
See specific comments below.
**Author response:** The text has been revised again, especially results section. Also, according to your previous comment, and because of the significant effect of the year × irrigation regime × cropping system on considered traits, the data of two years for barley and chickpea were presented, separately in tables and figures.

Abstract:
Line 25: “as split plot based on a randomized complete block design” I would remove this information from the abstract.

**Author response:** It's removed from the abstract
Line 37-30: I would suggest removing this detailed sentence from the abstract, it is more appropriate for materials and methods section. It is enough informative if you mention that the second factor was the sowing time (December vs January).

**Author response:** Cropping systems as sub plot consisted of sole and relay intercropping of barley with chickpea in two sowing dates (December vs January).

Line 31: “Chlorophyll a content of barley increased in b1c2, by consuming less water compared to sole cropping.” Better if you explain it in terms of…In case of relay intercropping, the early establishment of barley determined an higher chlorophyll content in biomass compared to… due to the… Please try to be more precise in your statement, why b1c1 consumed less water? Less competition from chickpea? Early establishment ensured a growth advantage and therefore a better use of water?
Line 33: What do you mean with “(b1c1 and b1c2) created a suitable canopy to pigment contents improvement”.

**Author response:** Under drought, the early establishment of barley in December intercropped with chickpea in January (b_1_c_2_) enhanced the chlorophyll content by 16% compared to sole cropping due to the less competition with chickpea.
Line 34 “carotenoid content of chickpea” please specify if you are referring to chickpea biomass or grains.

**Author response:** Late sowing of chickpea enhanced the leaf carotenoid content of chickpea, catalase and peroxidase activities.
Line 35-36 “Barley-chickpea intercropping reacted to water deficit through enzymes activity, water use efficiency and land equivalent enhancement compared to sole cropping”. The WUE and LER are not “reactions”. It is better saying that intercropping enhanced the WUE and guaranteed a more efficient use of space (LER>1) compared with sole crops.

**Author response:** Barley-chickpea intercropping enhanced the WUE and guaranteed a more efficient use of space (land equivalent ratio of more than 1) compared with sole crops.

Line 36-38 “Under water stress, in b1c2, by increasing total chlorophyll and water use efficiency, grain yield of barley enhanced compared to b1”. This sentence is not clear at all.

**Author response:** Under water stress, in b_1_c_2_ enhancement of total chlorophyll and water use efficiency caused to increase the grain yield of barley.

Line 38: “It seems”. Not appropriate for abstract

**Author response:** The barley and chickpea in b_1_c_2,_ responded to water stress by increasing total chlorophyll and enzymes activity, respectively.

Introduction:
My impression is that sentences are not linked to each other. The introduction needs to be rephased in order to improve the readability of the paper. Moreover, make clear your objectives and hypothesis. I strongly suggest submitting the manuscript for an extensive English revision by experts.

**Author response:** In introduction section, first the intercropping and relay intercropping systems were defined and then the phrases and topics were linked as below:

The importance of relay intercropping of legumes with cereals, the importance of barley and chickpea in Iran, the role water stress in the late season on crop production in semi-arid areas, the role of water stress in decreasing the photosynthetic pigments and RWC, and increasing enzyme activities. Finally, we describe the problems of crop production in Iran due to water stress and describe the hypothesis and objectives.

Line 104: carried out instead of “laid out”

**Author response:** It's done in the text.

Line 127: Is it Darab cv a registered cultivar of chickpea or an ecotype?

**Author response:** Darab cv is a registered cultivar.

Line 132: “which is suitable for hot and dry areas” better say suitable for arid/semi-arid conditions.

**Author response:** It's done in the text.

Line 183: “hand harvested manually” remove manually.

**Author response:** It's done in the text.

Material and methods:
Line 268-271: Please provide models that have been used, including fix and random parts. Moreover, did you check if 1) the responses for each factor level have a normal distribution, 2) If these distributions have the same variance and 3) if the data are independent? Otherwise, you should opt for gl(m)m assuming other data distributions. You just have 3 replications, were they enough for studying the interactions of your experiment? Were the residuals of your models normally distributed? You should add more information for this section. In particular I would suggest the author to include the structure of model that has been used (factor that you used in the analysis and if some factors were nested in others) and the procedure for model selection.

**Author response:** The model used for variables in analysis of variance was fix model for all variables including year, irrigation regime and cropping system. In order to check the normality distribution of data, Kolmogorov-Smirnov and Shapori-Wilk tests were used and the skewness and kurtosis indices of data proved that the distribution of data was normal. The F-test was carried out to check the equality of variances. The residuals of the model were also normal using q-q plot. By the way, most of the researchers use 3 replicates and is often enough for such field experiments. Data was analyzed by SAS software 2012 (version 9.4) and the means were compared by Tukey-Kramer test at 0.05 probability level (p≤ 0.05). Because of significant effect of year × irrigation regime × cropping system on considered traits, the data of two years for barley and chickpea presented, separately.

Results:

Line 300-314: I would move this part in MM section.

**Author response:** This part's moved to MM section.

Line 323: here and throughout the text use the same number of decimals for mean and SE.

**Author response:** It's done in the text.

Line 459-461: please, specify better that you are referring to the early established barley **Author response:** The early establishment of barley in December intercropped with late sowing of chickpea in January improved the competition ability of barley through the faster growth.

Line 461-463: This result is not clearly presented. Please rephase.

**Author response:** Under water stress in b_1_c_2_ treatment grain yield of barley was enhanced 19 and 35% compared to b_1_ in 2017 and 2018, respectively.

Line 468: Express yield as t ha-1 instead of kg ha^-1^.

**Author response:** It's done in the text and Figures.

Line 472-474: This is discussion and not results.

**Author response:** It's moved to discussion section.

Line 478: what is the biological yield. Add information on the procedure that you used for the determination of biological yield in MM section.

**Author response:** At the crop maturity stage on May, the plants in the central 1 m^2^ of each plot were hand harvested. Then, the samples were oven dried at 72 °C for 48 h and weighted for biological yield.

Line 478-493: Appropriate annotation should be used. t/ha instead of kg/ha. This section is almost impossible to read. The English should be significantly improved.

**Author response:** This section was revised.

Tables:
Table 1: “(mg/kg-1)” this is not the correct annotation. Please use mg/kg or mg kg-1. Moreover, you recorded N tot 0.75 mg/kg and it looks like very low. Check the measure unit, 0.75 g/kg seems more reasonable.

**Author response:** The unit's changed to mg/kg in table 1, but 0.75 mg/kg is correct according to soil test.

Table 3: what is the difference between “Year” and “replication (year)”

**Author response:** This is Replication × Year interaction.

Table 5 and 6: I would remove the capital letters. You mentioned that Year x Irrigation was significant so why did you perform the post-hoc test within years?

**Author response:** I think the previous comment of Reviewer #2 about the Tables was as:

'' Table 5: You mentioned that “year” and “irrigation regime” are significant factors. In this case you should perform the statistical analysis separately for each year and for each irrigation regime. In the table you have assigned letters of significance in the wrong way. Please perform a post hoc separately for year and irrigation regime and eventually assign capital letter for indicating differences between years. You should include standard error (SE).
Table 6: see comments above
Table 7: see comments above
Table 8: see comments above''

Therefore, because of the previous comment of Reviewer #2 in all of the tables, each irrigation regime and year were analyzed, separately and two years assign with capital letter for indicating differences between years.

I would suggest using some colors for increasing the readability of figures.
Reduce the number of figures. Information included in figures 3,4 and 5 can be presented in just one figure using colors and multiple bars.
Figure 1,2,3,4,5 and 6:
• Bars represent mean±SE instead of “Vertical bars represent ±SE”.

**Author response:** I think the previous comment of Reviewer #2 about the Figures was as: ''Figure 1: You should perform post-hoc of your model separately for 2017 and 2018. Use lowercase letter for 2017 and uppercase letter for 2018. SE are missing, please provide them. In case that year is not a significant factor you could present data as average between 2017 and 2018 for each of the treatments. Another option can be present data on 2017 and 2018 in two separate graphs''. Same for figure 2, 3, 4, 5, 6, 7.

Therefore, because of the previous comment of Reviewer #2 and significant effect of year on all of the traits and according to previous comment of Reviewer #2 in figure 1 and the other figures, data for each year were presented, separately.

“In each irrigation regime, means with a letter in common should not be considered different according to the Tukey's test at p≤0.05” this is not clear. I suggest to rephase as: Values with different letters are not significantly different at 0.05 confidence level.
Figure 3 and 4: Use t/ha instead of kg/ha

**Author response:** It's done in the text and Figures according to Editor's comment.

**Sincerely,**

**Corresponding author**
